# The Obese Brain—Effects of Bariatric Surgery on Energy Balance Neurocircuitry

**DOI:** 10.1007/s11883-015-0536-3

**Published:** 2015-08-25

**Authors:** José Carlos de Lima-Júnior, Lício A. Velloso, Bruno Geloneze

**Affiliations:** Laboratory of Cell Signaling, Department of Internal Medicine, University of Campinas—UNICAMP, Campinas, Brazil; Laboratory of Investigation in Metabolism and Diabetes—LIMED, University of Campinas, UNICAMP, 13084-970 Campinas, Brazil

**Keywords:** Obesity, Bariatric surgery, Roux-en Y gastric bypass, Brain, Hypothalamus

## Abstract

Obesity is a highly prevalent disease in the world and with a major impact on global health. While genetic components are also involved in its pathogenesis, in recent years, it has shown a critical role of the innate and adaptive immune cell response in many tissues triggered by excess of nutrients such as lipids and glucose. Free fatty acids and other nutrient-related signals induce damage such as insulin resistance in the peripheral tissues but also in the brain. Specifically in the hypothalamus, these metabolic signals can trigger significant changes in the control of energy balance. Recent studies have shown that saturated fat disrupts melanocortin signaling of hypothalamic neuronal subgroups pivotal to energy control. Bariatric surgery is a treatment option for obesity when other tools have failed, because it is more effective than pharmacotherapy concerning of weight loss itself and in improvement of obesity-related comorbidities. Here, we review the mechanisms by which Roux-en Y gastric bypass (RYGB) can change peripheral signals that modulate melanocortin circuits involved in the regulation of energy balance.

## Introduction

The central nervous system (CNS) orchestrates energy homeostasis. Adiposity signals, as leptin and insulin, gastrointestinal signals, and other stimuli feed itself reflect the body fat store, shuttling information to the brain, particularly the neuropeptidergic system in the arcuate nucleus (ARC) of the hypothalamus, which has been reported to modulate energy balance through melanocortinergic second-order neurons [1]. Food reward circuits act parallely with melanocortinergic circuitry in the management of the coordination of the energy requirements outputs that maintain a stable balance between spending and energy consumption [2].

Regardless of the intricate regulatory system of body fat reserves, more than one-third of adults in the United States are obese [3], resulting in an obesity pandemic that is reaching catastrophic proportions, impacting the incidence of a burden chronic disease and global mortality [4]. The hypothalamic alterations that result from exposure to high-fat diets and low-grade inflammation contribute to the pathogenesis of obesity and reorganization in the energy balance [5, 6••]. Thus, the brain changes that result from diet-induced obesity (DIO) affect brain programming to promote rescheduling in food intake, food interest, satiety signals response, and energy expenditure, and these changes contribute to a new energy balance set point in the obese brain [7, 8]. This new phenotype, which is characterized by a modified balance, implies a new defended level of adiposity that somehow contributes to the maintenance of obesity through the ARC. Signals from the ARC will deliver outputs to modulate the current adipose tissue stores and adjust to the obesity pattern through physiological and behavioral adaptations [9]. Increased adiposity is supported through decreased leptin/insulin signaling in the obese brain, which reduces the negative feedback of these adipostatic peripheral signals [1, 9]. As expected, the central administration of leptin has not been able to influence caloric intake [10]. Similarly, the anti-obesity drugs that are currently available mostly target the brain, but they are not optimally effective [11]. Therefore, bariatric surgery, which can be more effective to enhance the cerebral effects, is more effective as obesity therapy [12].

We herein will discuss the factors that are known to be involved in the neurobiological modifications are promoted by Roux-en Y gastric bypass (RYGB), and contribute to the reversal of the damage to the homeostasis neuronal circuitry, decreased food intake, and/or increased energy expenditure. An understanding of how the surgery is able to change the energy homeostasis set point in the obese brain of humans and rodents and partially modify the neuronal programming defense system of adiposity body is still incipient and will contribute greatly to the search for new therapeutic targets.

## Overview of the Hypothalamic Neuronal Systems for Energy Homeostasis

The central melanocortin system exerts control of expenditure and energy intake through neurons of the brainstem and two first-order neurons subpopulations located in the arcuate nucleus (ARC) of the hypothalamus, which transmit information on metabolic status—pro-opiomelanocortin (POMC) and neuropeptide Y/agouti-related protein (NPY/AgRP) neurons [11]. POMC neurons comprise two distinct γ-aminobutyric acid (GABA)-ergic and glutamatergic neuronal subpopulations that express the anorexigenic peptides POMC [α-melanocyte stimulating hormone (MSH)] and cocaine- and amphetamine-related transcript (CART), whereas AgRP neurons express the orexigenic peptides NPY, AgRP, and neurotransmitter GABA, although the existence of these neurotransmitters is controversial [13, 14]. Other system components are second-order neurons that express the melanocortin receptors MCR3 and MCR4 and that receive ARC inputs [13] (see [15] for a review). POMC- and CART-positive neurons and NPY/AgRP, respectively, send projections within the hypothalamus to the periventricular nucleus, paraventricular nucleus (PVN), perifornical area (PFA), and lateral nucleus (LH), as well to the brainstem [16, 17]

These two neural ARC subpopulations are targeted by several signaling peptides, including insulin, leptin, and gastrointestinal hormones, such as CCK, peptide YY, ghrelin, and glucagon-like peptide-1 (GLP), which, except for the unique gastrointestinal orexigenic hormone, ghrelin, have anorexigenic effects on POMC [18, 19]. During the fed state, there is an increase in leptin and insulin levels in proportion to fat store, in order to stimulate the transcription of anorexigenic POMC peptides and decrease the expression of orexigenic peptides NPY and AGRP in order to reduce food intake and increase energetic expenditure, this mechanism makes up the peripheral adipostatic system [15, 20, 21].

## How Fatty Acids Affect the Hypothalamus and Deregulates Energetic Homeostasis

Insulin resistance is the main metabolic change that is associated with obesity and the chronic inflammation of adipose tissue, and macrophage activation in this tissue plays a crucial role in this disease mechanism [22]. These findings illustrate the interconnection between the lipid signaling, inflammation, and energy homeostasis [23]. Saturated fatty acids, which derive from the diet and are crucial components in the pro-inflammatory response, activate inflammatory signaling in various tissues through TLR4 activation and the subsequent stimulus of the inhibitor of Kβ (IKKβ) and nuclear factor κβ (NFκβ) pathways, such that this process culminates in blocking the transduction of the insulin and leptin signals in these tissues [24]. It is not surprising that this process also occurs in the hypothalamus and affects energy homeostasis [25]. Because leptin and insulin have a central role in adipostatic signaling, a deterioration in their action in the hypothalamus could initiate a new set point for energy in an adaptive process that favors energy intake and subsequent weight gain [26]. High fat feeding has been involved in several important processes that affect the neural physiology of the melanocortin system [27]. Thus, the state of insulin and leptin can affect the firing of the neuronal subpopulations that are crucial in energy balance [28]. Likewise, in rats fed with saturated fatty acids, the Toll-like receptor 4 acts as a molecular trigger for inflammatory signaling in the hypothalamus, thus impairing the anorexigenic signals that maintain the energy balance [29]. Additionally, high-fat feeding triggers apoptosis and major changes in synaptic plasticity in hypothalamic neurons [30].

## Evidence from Human Observational Studies That RYGB Affects the Hypothalamus

The unavoidable question is whether the surgical weight loss causes regression of the neuronal damage that is induced by obesity and if such hypothalamic changes alter the neural control and reset the set-point energy balance.

A number of neuroimaging studies of the effects of RYGB on eating behavior have shown the involvement of the brain’s the reward system. A reduction in the activation of these areas in response to food cues after RYGB has mapped the changes in limbic circuitry of hedonic drive. However, in addition to the impact on the mesolimbic system, other studies have demonstrated that RYGB results in major changes in the regulation of energy expenditure and that these changes are fundamental for maintaining the negative balance [31•].

The hypothalamus plays a central role in regulating this homeostasis. Matsuda et al. demonstrated for the first time in humans an anomaly in the hypothalamus and a difference between lean and obese individuals that could correspond to central damage that is caused by obesity. They performed functional magnetic resonance imaging (fMRI) after oral glucose intake and demonstrated that obese individuals exhibit an attenuation and delay of the fMRI signal in the ventromedial nucleus (VMH) and PVN in the hypothalamus [32]. Similarly, Thaler et al. showed that obese subjects had evidence of gliosis in their mediobasal hypothalamus (MBH), which was associated with body mass index (BMI). Interestingly, the correlation between BMI and signal intensity was restricted to the hypothalamus [6]. Additional studies have been published that support these findings and that shed light on the effects of RYGB on neural control (Table 1). Frank et al. [33] demonstrated in a cross-sectional study that changes in corporal weight after RYGB are capable to induce changes within the hypothalamus. Severely obese women presented greater activation during the presentation of low-calorie food and lower hypothalamic activation during the presentation of high-calorie food compared with normal-weight women and RYGB women. In contrast, the RYGB women exhibited a hypothalamic response that was analogous to those of normal-weight women and distinct from those of severely obese women, thus demonstrating a normalization of hypothalamic brain activity after RYGB.

**Table 1:** Evidence of RYGB weight loss effects on hypothalamic neuronal responsiveness in humans—neuroimaging studies

Reference	Individuals	Design/methods	Results	Commentary
Frank et al. [33]	Nine severely obese women who had undergone RYGB	Functional resonance magnetic imaging was performed to analyze hunger and satiety while food and non-food pictures were presented to three different groups: obese women vs normal weight women vs women who had undergone RYGB	Obese women showed increased hypothalamic activation during the presentation of low-calorie food pictures and lower activation during exposition for high-calorie food pictures, which was the opposite model of normal weight and operated groups	Although the study does not allow conclusions about hypothalamic mechanisms involved in satiety, the results showed similar hypothalamic activation in the RYGB group compared to normal-weight, which could indicate attenuation of a neural impairment
Rachid et al. 34	Twelve obese non-diabetic individuals undergoing RYGB and lean controls	Preoperatively and 8 months postoperatively, the individuals were evaluated for hypothalamic activity with fMRI after exposure to cold in order to study the involvement of central hypothalamic activation in the recruitment of brown/beige adipose tissue that was detected by 18F-FDG PET/CT-scan and real-time polymerase chain reaction measurements of signature genes of brown/beige and white adipose tissue	After weight loss, higher metabolic activity of brown/beige adipose tissue was detected by PET/CT and biopsy, but this change occurred independently of the hypothalamic activity	Unprecedented assessment of browning after RYGB and its regulation by the hypothalamus. There is experimental evidence that leptin and insulin lead to greater browning through the regulation of POMC neurons in the arcuate nucleus. The improvements in the signaling of these hormones in the hypothalamus with weight loss induced by RYGB would increase BAT activity, which was not measurable in this paper
van de Sande-Lee et al. [36]	Thirteen obese and non-diabetic patients underwent RYGB and lean controls	Preoperatively and 8 months after RYGB, these patients underwent a temporal clustering analysis (TCA) and functional connectivity MRI (fcMRI) after the ingestion of 50 g of d-glucose	With a TCA, it is possible to evaluate the signal activity at an anatomic location, and MRI indicated that lean and obese patients presented different presurgical activities in the hypothalamus. These activities were more similar between lean individuals and obese patients after surgery. For fcMRI, the postsurgical obese patients exhibited the highest functional connectivity between the hypothalamus and the orbitofrontal, somatosensory, and orbital cortices	The changes after massive weight loss normalize toward the well-know patterns in lean brain, which is important evidence that obesity-induced hypothalamic dysfunction may be reversible and a potential target for new drugs

In another study, Rachid et al. [34] explored the central regulation of brown adipogenesis in humans based on the finding that brown adipose tissue (BAT) in rodents is controlled by hypothalamic sympathetic outputs. They evaluated the hypothalamic response in humans after a cold stimulus, because brown/beige adipose tissue is mainly recruited in this situation. They wondered how weight loss that was induced by RYGB would affect the activation of BAT and if the recruitment was due to changes in the hypothalamic neural activation [35]. Their results showed that significant weight loss resulted in greater activation of BAT, which was not accompanied by changes in hypothalamic neuronal activity. These findings suggested that the damage that was induced by obesity in the brain region that controls whole-body energy homeostasis might be irreversible or only partially reversible in humans. The same group demonstrated the partial reversibility of hypothalamic damage after surgical weight loss with fMRI of brain activity after glucose ingestion. Thus, the structural changes that occur in the postsurgical obese brain bring it closer to the template of a lean brain. Another important finding was an increase of interleukin (IL)-6 and IL-10 in the cerebrospinal fluid (CSF) of these obese individuals after surgery [36]. Interestingly, these findings have suggested that the attenuation of inflammation markers in the CSF correlated with the changes in brain activity as well as the increase in the levels of anti-inflammatory cytokines, thus suggesting a route for mechanistic investigations in humans [36].

## Mechanistic Insights Underlying the Effects of RYGB on the Hypothalamic Circuitry

**Fig. 1:**
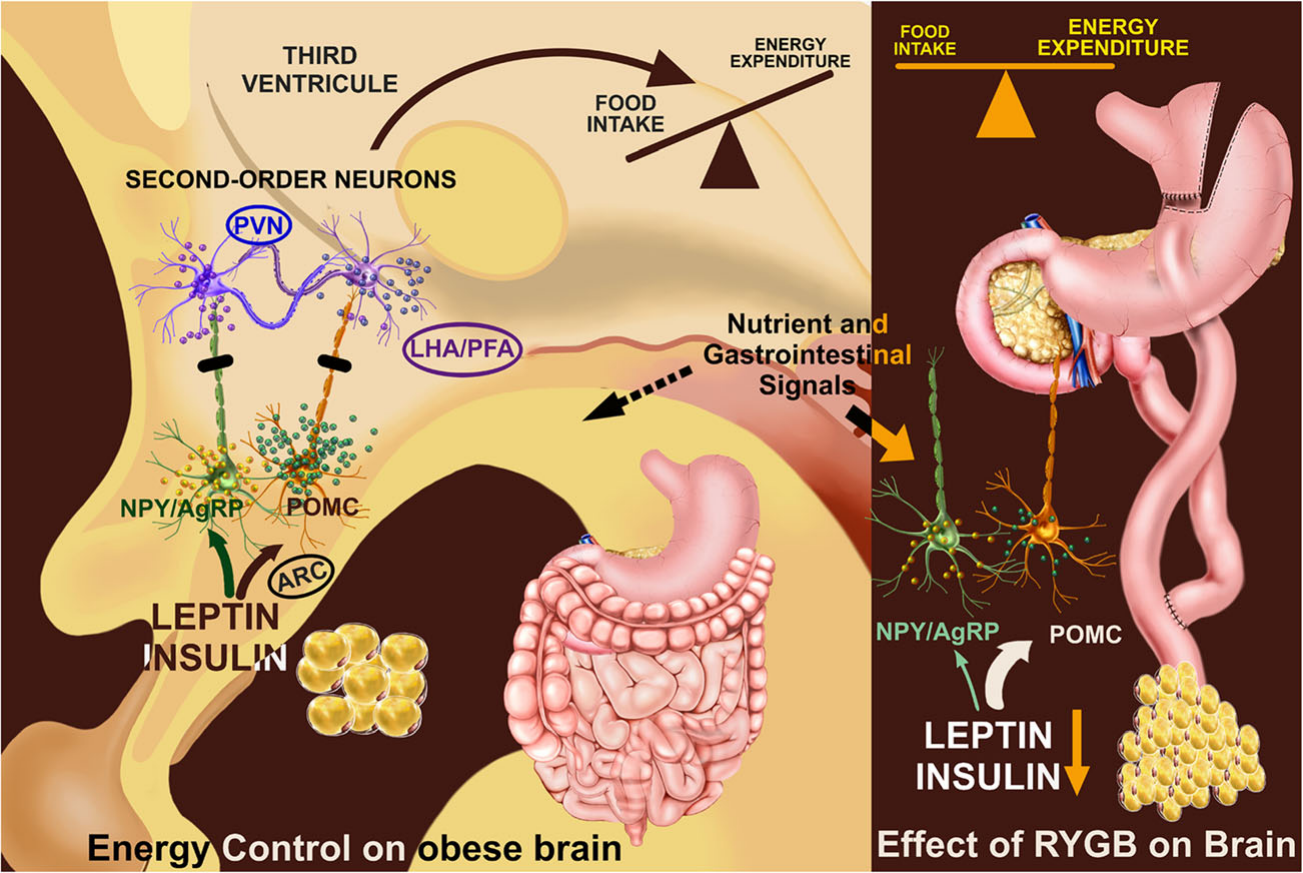
The effects of RYGB on the main hypothalamic circuitry that controls energy balance. On the *left*, first-order neurons in the ARC nucleus in an obese state. This panel shows a simplified circuit of these neuronal sensing of peripheral signals that regulate energy balance. During the obese state, hypothalamic leptin and insulin resistance occur. Thus, downstream activation of second-order neurons (PVN, LHA/PFA) to reduce food intake and lead to higher energy expenditure is impaired. On the *right*, RYGB results in a possible change in central regulation by adipostatic signals leptin and insulin, in a higher secretion of anorexigenic peptides, such as PYY and GLP-1, and bile acids. Such modifications through mechanisms not fully demonstrated have targeted the hypothalamic center of energy regulation

RYGB is an effective option for treatment of obesity, type 2 diabetes, and insulin resistance. However, which mechanisms are involved is still not very clear, and these may also cause changes in the energy expenditure in the central nervous system or in the gut-brain communication (Fig. 1) [37].

### Leptin and Insulin as Protagonists

As mentioned previously, leptin and insulin signaling in the hypothalamus is crucial for the regulation of energy balance on the melanocortin system [38, 39]. In obesity, the serum levels of these hormones are increased in parallel with the resistance to receptor-mediated signaling [7]. Defective insulin and leptin signaling in the hypothalamus prevents input to the anorexigenic areas by adiposity negative feedback, thus enhancing the food intake [40].

Because RYGB induces profound changes in several factors that are involved in energy homeostasis signaling, it has been hypothesized that such changes will be able to partially restore the lean set point of energy balance. However, such beneficial changes that would cause weight loss are extremely difficult because the physiological compensatory changes occur in energy expenditure in order to oppose the variation in the weight to maintain the usual weight. [41]

Recently, it was described in human BAT activity, which is located primarily on cervical and supraclavicular depot. Brown fat produces heat though thermogenesis induced by mitochondrial UCP1 and is important for energy expenditure in the defense against cold and obesity [42, 43]. Recently, BAT has emerged as a potential therapeutic opportunity [44]. These functions suggest that low or no function of brown/beige adipose tissue could cause a propensity to DIO [45, 46].

The central route for the control of metabolorregulatory thermogenesis could be confused with feeding behavior and the thermoregulatory pathway [47]. Signals in the blood, such as insulin, leptin, CCK, enterostatin, GLP1, adenosine, serotonin, endocannabinoids, angiotensin, α-MSH, and ghrelin can act in nutrient-sensing areas, such as the ARC, or directly in VMH to regulate BAT activation [48]. For instance, leptin and insulin act together on POMC neurons to drive browning of white adipose tissue, which culminates in weight loss and increased energy expenditure [49]. Thus, the hypothesis that the enhancement of brown/beige adipose tissue activity after RYGB occurs through hypothalamic regulation, as demonstrated by Rachid et al. [34] may be a result of improved insulin and leptin sensitivity on the hypothalamus after weight loss is plausible.

To assess the importance of improving insulin sensitivity in hypothalamic signaling after RYGB, it was obtained a knockdown animal model for the insulin receptor kinase domain in the VMH (VMH IRkd). After RYGB, these Sprague-Dawley rats exhibited significant impairments in hepatic glucose production during hyperinsulinemic-euglycemic clamp versus sham RYGB. These data suggest that an improved sensitivity to insulin in the VMH might be one of the mechanisms underlying the amelioration of glucose homeostasis after surgery, which was modulated in the liver. The observation that the postsurgical insulin-induced glucose disposal was not completely improved in the knockdown rats suggested that other pathways and regions might be involved in the metabolic benefits of the procedure [50••].

The effects of insulin action on neuronal activity in the brain can be investigated by fMRI in humans by using blood-oxygen-level-dependent (BOLD) contrast imaging or cerebral blood flow (CBF) after insulin or glucose injection [51]. Using this method, van de Sande-Lee et al. demonstrated changes in neuronal activity in the hypothalamus after RYGB with an fMRI protocol that evaluated hypothalamic connectivity after the oral ingestion of 50 g d-glucose [36].

In addition to insulin levels, leptin levels decrease after RYGB [52]. This also suggests the central question: does RYGB able to reset the leptin/insulin adipostatic set point in the hypothalamus? [53] As mentioned above, the weight loss changes occur in order to defend the level of adiposity prior to weight loss. After the surgery, leptin levels decrease over time [54], which could stem from the improvement in leptin signaling in the body or an energetic adjustment that is made to maintain pre-weight loss state through reduced energy expenditure and increased food consumption. This seems more reasonable in view of the weight regain rate after dieting [55] or even after surgery [56, 57]. We will not be able to exactly answer the question about the resetting of the set point. Although the neuronal cells are endowed with plasticity and evidence of the reversibility of the brain damage has been reported, even in humans (Table 1) [58], leptin continues working to keep it that way once the individual is obese [59]. This relative leptin insufficiency that occurs in parallel with the weight reduction induces weight regain because its central action is mirrored in key areas of eating behavior and energy homeostasis, and this action is therefore reversible when the weight loss is accompanied by replacement leptin [60]. In leptin deficient ob/ob mice, RYGB does not produce weight loss. Initial weight loss is soon followed by restoration of the weight to presurgical levels, and this is partially corrected with replacement leptin [61]. This reversibility of leptin resistance is due to plasticity in the ARC melanocortinergics neurons, which is promising for the maintenance of the profound changes that are caused by RYGB. When extrapolating to knifeless weight loss in mice, an energy-restricted diet simultaneously promotes decreased levels of leptin and simultaneously promotes increased expression of neurons NPY/AgRP and does not enhance the activation of POMC neurons, which puts this neural recovery in check [62, 63]. In humans, a clinical trial of the administration of leptin after RYGB in women with relative hypoleptinemia after surgery did not have beneficial effects on body composition or energy expenditure [64•].

The current knowledge about whether this set point is resettable or not comes from animal models, and absolute studies are lacking. Sprague-Dawley rats that underwent RYGB showed 10 days after surgery decreased NPY-immunoreactive neurons and increased α-MSH-immunoreactive neurons in the ARC, parvocellular PVN, and magnocellular PVN [63]. A decrease in NPY receptors in the PVN indicates increased NPY signaling activity as opposed to decreased POMC signaling. These findings indicate that the restriction of energy intake results in a hungry obese brain rather than in a newly satiated thin brain [53]. Apparently, when we compare Sprague-Dawley rats’ DIO with a successful RYGB (RYGB-S) and those with a failed bypass (RYGB-F), RYGB-S rats exhibit increased expression of the leptin receptor in the hypothalamus compared to the other group, which exhibits reduced serum leptin levels, most notably in the RYGB-S. The most logical explanation is that there is no change in the set point hypothalamic leptin, insofar as the greatest weight loss might have occurred due to compensatory catabolic changes that were promoted by the increase of PYY in these rats. PYY expression was also increased in the hypothalamus, followed by an inhibition of NPY/AgRP and POMC/CART expression was increased. This study of the characteristics of the underlying mechanisms also noted some changes in other candidates that are involved in gut-brain axis [65].

### Candidate Signals Operating in the Gut-Brain Axis

The obvious candidates for involvement in the new regulation of energy balance after RYGB are peptides that are secreted by the enteroendocrine cells in the gastrointestinal tract, are involved in the regulation of food intake, have nutrient-sensing mechanisms, and act in the hypothalamus or hindbrain. Thereby, an anatomical change in the gastrointestinal tract must change their secretion, and why not optimize its signaling in the hypothalamus to enhance the satiety? (See [18] for a review).

Most studies have reported an increase in the secretion of anorexigenic peptides after RYGB—such as PYY, GLP-1, amylin, oxyntomodulin, CCK, and total bile acids (TBA), and the only one with orexigenic effect, ghrelin, exhibits more complex behavior but with generally reduced levels [66–71]. This enhanced response is persistent and supports weight loss, despite the presurgical-defended level of adiposity controlled by leptin [72].

Currently, other factors have been shown to be involved in the mechanistic roles underlying RYGB’s effects on the brain, such as changes in the intestinal microbiota and signaling by bile acids through the farsenoid-X receptor (FXR) and TGR5 receptor membrane [53, 73]. Although there is an abundant negative correlation between body weight after RYGB and postoperatively increased levels of gastrointestinal peptides, the literature is more saturated when it comes to GLP-1 and PYY, and direct mechanistic evidence is partially missing. The central signaling of GLP-1R does not seem to have a pivotal role in downstream cascade that results in postsurgical weight loss, which is a phenomenon that was confirmed by the similar amounts of weight loss between GLP-1R-knockout (KO) mice and wild-type mice that underwent RYGB [74••]. Similarly, although the GLP-1 leads to BAT thermogenesis after its central administration [75], its peripheral levels are not able to increase energy expenditure after RYGB in rats [76]. After performing a modified gastric bypass in PYY-KO mice, the effects of weight loss were lost, thus establishing a critical role of this hormone in weight loss mechanisms. [77]

Recently, Fang S et al. demonstrated that administration of gut-restricted agonist FXR leads to weight loss, browning, and increased thermogenesis through an increase in fibroblast growth factor 15 (FGF15) [78]. Thus, if the control of BAT thermogenesis and browning occurs centrally and involves GLP-1R signaling in the brain [75] and if there are TGR5 receptors for bile acids in the brain [79], crosstalk between GLP-1R and TGR5 brain receptor signaling is possible. This crosstalk would contribute to greater energy expenditure through RYGB-induced thermogenesis because this surgery involves changes in the delivery of bile acids into the modified gastrointestinal tract.

## Concluding Remarks

In recent years, the increase in obesity has led to substantial increase in the study of its important clinical and pathophysiological aspects, and scientific efforts have more recently tried to clarify the molecular mechanisms underlying the changes in energy balance and, particularly, the changes in hypothalamic circuitry, which are key to the evolution of pharmacotherapy. These scientific advances have contributed to a description of the installation of the hypothalamic injury as well as to how the surgery can modify the programming brain and drive it to a favorable energy balance. However, there are still many gaps in the understanding, and new avenues are opening up every day. Although RYGB has important effects on the population, including reduction in cardiovascular mortality, an in-depth understanding of how and why the surgery causes such profound changes, particularly in this hypothalamic neuronal group that regulates energy homeostasis, is lacking.
